# Tunable and unconventional Fermi arcs of two-dimensional transition-metal dichalcogenide modulated photonic Dirac semimetal

**DOI:** 10.1515/nanoph-2025-0083

**Published:** 2025-06-11

**Authors:** Yang Yang, Hongye Qiu, Ke Bi, Biao Yang

**Affiliations:** State Key Laboratory of Information Photonics and Optical Communications, School of Physical Science and Technology, 12472 Beijing University of Posts and Telecommunications, Beijing 100876, China; College of Advanced Interdisciplinary Studies, National University of Defense Technology, Changsha, China

**Keywords:** metamaterial, surface wave, topological photonics

## Abstract

Fermi arcs are nontrivial surface states that exist in topological semimetals, which exhibit a variety of interesting effects, such as anomalous transport properties and chiral anomaly induced phenomena. Recently, the emerged Two-dimensional transition-metal dichalcogenide (TMDC) shows distinctive optical and electrical properties, makes it a promising platform for efficient modulation of Fermi arcs. By covering TMDC sheets on a photonic Dirac metamaterial (PDS), the quadrupole Dirac point splits into two triple degeneracy points (TDPs), each TDP share one Fermi arc. Through tuning the characteristics of TMDC layers, Fermi arcs and transmissions of PDS can be effectively modulated in multi-degrees of freedom. Unconventionally, we find the Fermi arcs may do not terminate at the degeneracy points but between the two type III TDPs. Fermi arcs with nonlocal effect are also investigated. Furthermore, topological transition from open (hyperbolic-like) to closed (elliptical-like) equi-frequency contours at TDP is also observed. Our findings may provide potential applications in flexible modulation of Fermi arcs with multiple functions.

## 1 Introduction

Study on topological phases of matter has achieved substantial and extensive progress over the last few decades. The most important characteristic of topological materials is the existence of nontrivial surface waves at the interface between two media with different topological charges [1], [2]. Fermi arcs are one type of nontrivial surface state exist in certain materials such as Weyl semimetal or Dirac semimetals, where a portion of the Fermi surface is truncated that forms an arc-like structure [3], [4], [5], [6]. The characteristics of Fermi arcs determines the propagation properties of topologically protected surface waves, leading to a variety of interesting effects, such as anomalous transport properties, negative-index flat lenses in photonic systems and chiral anomaly induced phenomena [7], [8], [9], [10], [11], [12], [13], [14], [15]. Such that multiple modulation of Fermi arcs is essential to promote its further application.

Recently, the layered transition metal dichalcogenides (TMDC) have attracted extensive attention for their novel physical properties in reduced dimension [16], [17], [18]. Monolayer TMDC has a large direct band gap, which is conducive to the production of photoelectric devices with excellent performance. Furthermore, monolayer TMDC possesses tightly bound neutral and charged excitons and display strong excitonic properties, providing them as a unique platform for applications in optoelectronics as light emitters [19], field effect transistors [20], spintronics integration [21] and nanophotonics [22]. These unique electronic and optical features make them promising for efficient modulation of Fermi arcs.

## 2 Methods

**Figure 1:**
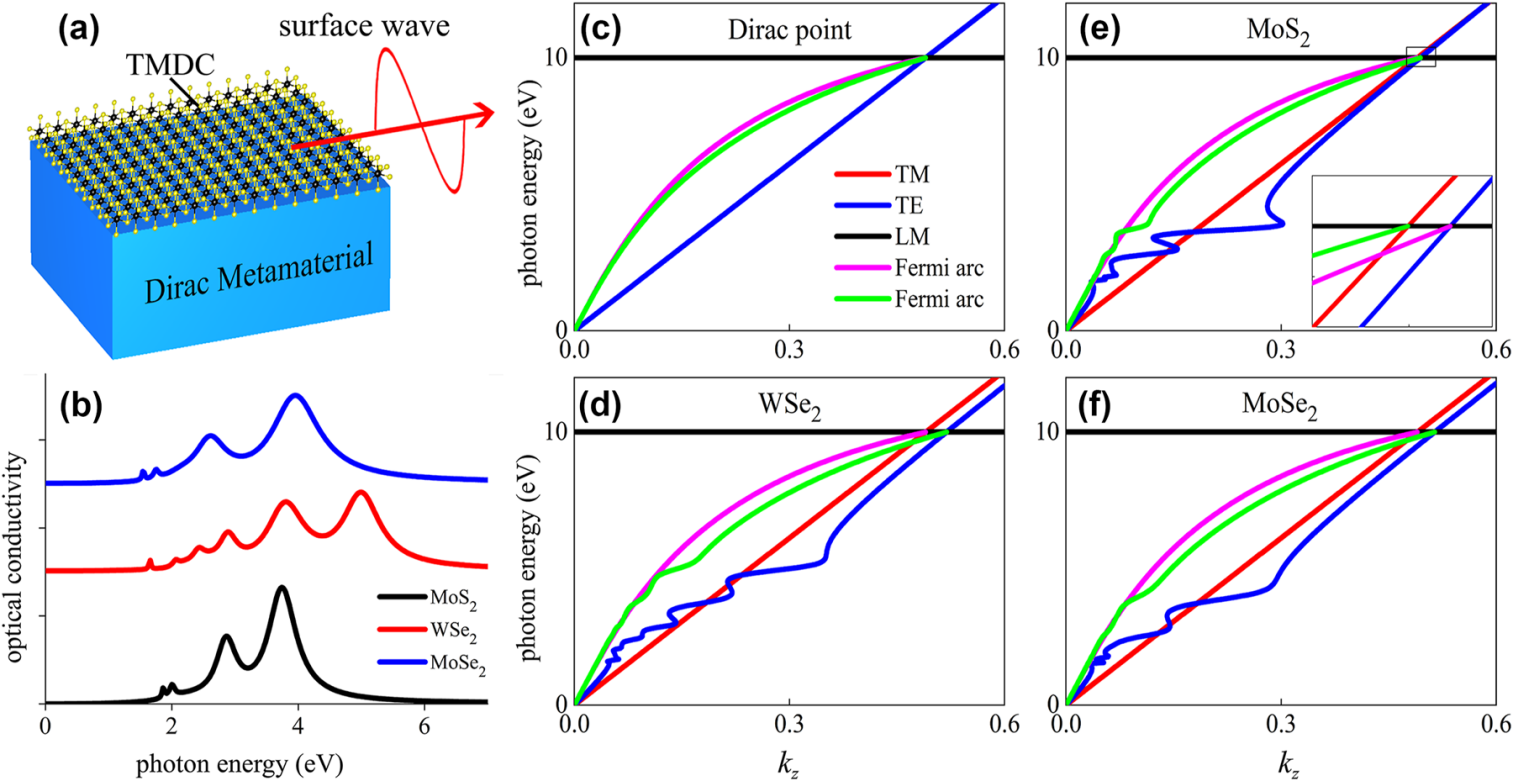
Modulation of Fermi arcs of DP with TMDC monolayer. (a) Schematic diagram: TMDC sheets covered on PDS to manipulate Fermi arc surface state; (b) real part of the optical conductivity of TMDC monolayer: MoS_2_, WSe_2_, MoSe_2_, respectively. Band structure and Fermi arcs of (c) PDS; (d)–(f) PDS with covered TMDC monolayer: (d) WSe_2_, (e) MoS_2_, (f) MoSe_2_, respectively.

Schematic of the configuration is shown in Figure 1(a): various TMDC sheets are covered on a three-dimensional photonic Dirac semimetal (PDS) to modulate the Fermi arc surface state. Optical conductivity *σ*(*ω*) of TMDC monolayer materials can be described by a superposition of Lorentzian functions [23]. Figure 1(b) shows the real part of the optical conductivity of MoS_2_, WSe_2_ and MoSe_2_ monolayer that normalized in unit of *σ*_0_ = *e* ^2^/4*ℏ* (universal dynamic conductivity of graphene) for the photon energy 1.5–7 eV, respectively.

Constitutive parameters of permittivity and permeability of PDS can be expressed by $\varepsilon =\left\{\varepsilon_{1},\varepsilon_{1},\varepsilon_{z}\right\},\mu =\left\{\mu_{1},\mu_{1},\mu_{z}\right\}$ [10]. Band structure and Fermi arcs of the Dirac point (DP) are shown in Figure 1(c), the linear crossings of two longitudinal modes (LMs) and two transverse modes (TMs) forms the quadruple degenerate DP with topological charge 2 is located at $k_{z}=\omega_{p}\sqrt{\varepsilon_{1}\mu_{1}}$ , which is protected by electromagnetic duality symmetry and all bands are double degenerate. At the plasmon frequency (*ω* = *ω*_p_), there are two Fermi arcs terminating at DP. The two Fermi arcs support spin-polarized one-way propagation, that each Fermi arc contributes to one Weyl point and they are mutually orthogonal with each other and the two Fermi arcs cannot be scattered into each other.

By covering TMDC sheets onto PDS, *C*_4_ rotation symmetry of PDS is broken into *C*_2_, such that the quadruple degeneracy DP splits into two type III triple degeneracy points (TDPs), where the location of one TDP that formed by the degeneracy of TM and two LMs is not changed; the other TDP that formed by the degeneracy of TE and two LMs shifts to $k_{z}=\sqrt{\varepsilon_{1}\omega_{p}-i\sigma \left(\omega \right)}\sqrt{\mu_{1}\omega_{p}}$ . Following the approach developed by Dyakonov [24], dispersion of surface wave supported by TDP (interface between air and TDP medium) is obtain as (Appendix A)

(1) $$\begin{array}{cc} & k_{z}^{2}\sqrt{\mu_{1}}\left(\omega_{p}^{2}-\omega^{2}\right)\sqrt{i\omega \left(\sigma \left(\omega \right)+i\omega \varepsilon_{1}\right)}\\ & \;=\omega \left[\begin{array}{c} \sqrt{k_{z}^{2}-\varepsilon_{1}\mu_{1}\omega^{2}}\left(i\omega^{2}\sqrt{k_{z}^{2}+\mu_{1}\omega \left(i\sigma \left(\omega \right)-\varepsilon_{1}\omega \right)}+\sqrt{kz^{2}-\omega^{2}}\sqrt{\omega^{2}-\omega_{p}^{2}}\sqrt{i\omega \left(\sigma \left(\omega \right)+i\omega \varepsilon_{1}\right)}\right)\\ +\sqrt{\mu_{1}}\omega \left(i\sqrt{k_{z}^{2}-\omega^{2}}\sqrt{\omega^{2}-\omega_{p}^{2}}\sqrt{k_{z}^{2}+\mu_{1}\omega \left(i\sigma \left(\omega \right)-\varepsilon_{1}\omega \right)}-\left(\omega^{2}-\omega_{p}^{2}\right)\sqrt{i\omega \left(\sigma \left(\omega \right)+i\omega \varepsilon_{1}\right)}\right) \end{array}\right] \end{array}$$

## 3 Results and discussion

**Figure 2:**
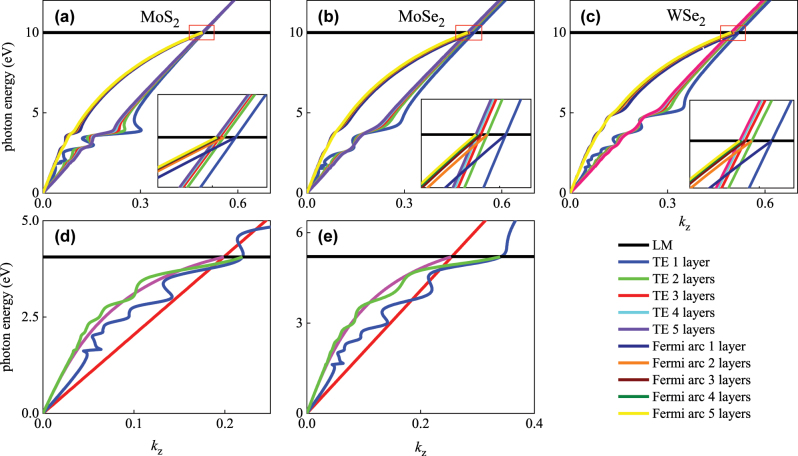
Tunable TDPs and Fermi arcs with the variation of number of layers (1–5) of the covered TMDC sheets: (a) MoS_2_, (b) MoSe_2_, (c) WSe_2_; (d)–(e): with the variation of LM of PDS with covered WSe_2_ monolayer.

Band structure and Fermi arcs of TDPs that generated by the coupling between the TMDC monolayer and PDS are respectively shown in Figure 1(d) WSe_2_, (e) MoS_2_, (f) MoSe_2_. It shows each TDP occupies one single Fermi arc, and the TE band and its corresponding Fermi arcs of TDP exhibit significant oscillating features that originates from the exciton transition that attributed to the splitting of the valence band by spin–orbit coupling of covered TDMC layers. The inset in Figure 2(e) shows the detailed characteristics of band structure and Fermi arcs of TDPs.

Fermi arcs can be further modulated by tuning the number of layers of the covered TMDC sheets on PDS. Optical conductivity of the multi-layer TMDC sheets can be expressed by $\sigma_{m}\left(\omega \right)=\sigma \left(\omega \right)/n$ , where *σ*(*ω*) is the complex optical conductivity of monolayer TMDC and *n* is the number of layers. Figure 2(a)–(c) show the variations of Fermi arcs by tuning the number of layers (1–5) of TMDC sheets: (a) MoS_2_, (b) MoSe_2_, (c) WSe_2_. As shown in the figure, by tuning the number of layers of TMDC, Fermi arcs of surface wave can be continuously modulated over a wide range. Since the surface conductivity of TMDC sheets is inversely proportional to the number of layers, locations of TDP move to a smaller wavevector *k*_*z*_ with the number of layers increasing, the corresponding Fermi arcs simultaneously shift, while the oscillations of Fermi arcs weaken gradually. The insets show the detailed transformation of TDPs and Fermi arcs: it shows that with the number of layers of TMDC increasing from 1 to 5, displacements of TDPs and Fermi arcs decrease gradually and eventually becomes stable.

Locations of TDPs and corresponding Fermi arcs can also be tuned by modifying the LMs of PDS, which can be adjusted by tuning the structural parameter of unit cell of PDS. As shown in Figure 2(d) and (e), by tuning the LMs to lower energy, Fermi arcs can be modulated into the oscillating region of the covered WSe_2_ monolayer and separations between the two TDPs can also be adjusted. Therefore, Fermi arcs of DP system can be flexibly controlled by the 2D TMDC materials in multiple degrees of freedom.

We further investigate Fermi arcs distributions in the *k*_*x*_−*k*_*y*_ plane with the variation of frequency around TDP. Without loss of generality, we consider the general TDP that splitting from the quadruple degeneracy DP with the constitutive parameters of electromagnetic system as $\varepsilon =\left\{\varepsilon_{x},\varepsilon_{y},\varepsilon_{z}\right\},\mu =\left\{\mu_{1},\mu_{1},\mu_{z}\right\}$ . Effective Hamiltonian of such TDP can be obtained by using the *k·p* method as (Appendix B)

(2) $$H_{\text{eff}}=c_{1}k_{z}I+\left(\begin{array}{ccc} c_{2}k_{z} & -\frac{ik_{x}}{\sqrt{\varepsilon_{z}\mu_{1}}} & -\frac{ik_{y}}{\sqrt{\varepsilon_{x}\mu_{z}}}\\ \frac{ik_{x}}{\sqrt{\varepsilon_{z}\mu_{1}}} & -c_{2}k_{z} & 0\\ \frac{ik_{y}}{\sqrt{\varepsilon_{x}\mu_{z}}} & 0 & -c_{2}k_{z} \end{array}\right)$$

where $c_{1}=c_{2}=1/\sqrt{\varepsilon_{x}\mu_{1}}$ , such that the TDP is a type III TDP, which is formed by a linear crossing between a doubly degenerate band (Weyl degeneracy) and an LM. The type III TDP here does not have a well-defined topological charge (winding number = 0), as there does not exist a fully gapped sphere surrounding TDP in the Brillouin zone (BZ) [25], [26], [27], [28], [29].

**Figure 3:**
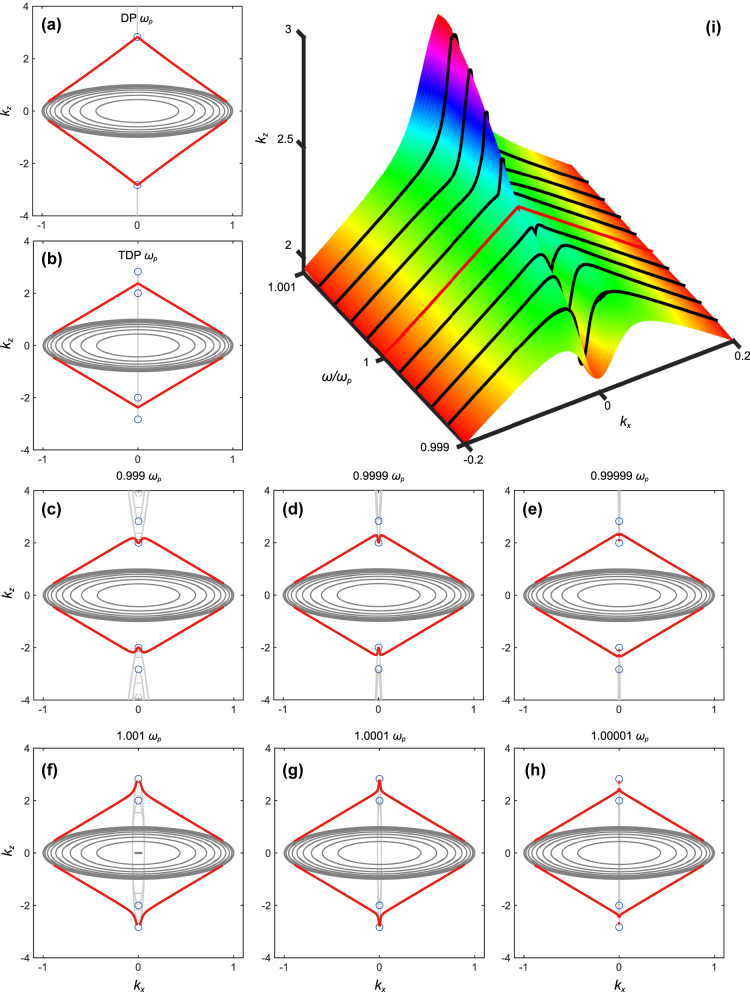
Band structure and corresponding Fermi arcs in the *k*_*x*_ − *k*_*z*_ plane (a) DP and (b) TDP; the black lines indicate the projected isofrequency surface (bulk states). Variation of Fermi arcs with the frequency approaching the type III TDP: (c)–(e) below the TDP frequency; (f)–(h) above the TDP frequency. (i) 3D diagram of Fermi arcs (black lines) variation as frequency approaches TDP. The red line in the middle shows the Fermi arc right at *ω*_TDP_.

Numerical calculations are shown in Figure 3, where the two type III TDPs indicated by blue circles respectively locate at $\omega =\omega_{p},k_{z}=\sqrt{\varepsilon_{x,y}\mu_{1}}\omega_{p}$ (*k*_*x*_ = *k*_*y*_ = 0), and the black lines indicate the projection of equi-frequency contours (EFCs). It is shown at TDP frequency in Figure 3(b), an endpoint of Fermi arcs (red lines) is tangential to EFC, and the other endpoint do not connect to any of the two type III TDPs, just locate at the space between them. This phenomenon is unconventional, since in general, the endpoint of Fermi arc should terminate at the degeneracy point with opposite topological charge, as is shown in Figure 3(a), the two Fermi arcs with opposite chirality both terminate at DP.

To unveil the underlying mechanism, we further investigate transformation of Fermi arcs around TDP frequency, Figure 3(c)–(e): approaching TDP from the frequency below TDP; Figure 3(f)–(h): approaching TDP from the frequency above TDP. It shows below the TDP frequency, Fermi arcs connect to TDP 1 (smaller *k*_*z*_), while above the TDP frequency, Fermi arcs connect to TDP 2 (larger *k*_*z*_). But when the frequency is very close to TDP, very interestingly, the endpoints of Fermi arcs do not know which TDP to connect, hence instead of directly linking to the two TDPs, they just terminate between them. This indicates the presence of the unconventional Fermi arc may be induced by the non-dispersive LM of type III TDP. In type III TDPs, the crossing bands exhibit a non-dispersive longitudinal mode along specific high-symmetry directions. As frequency varies, the endpoints of the Fermi arcs shift to remain consistent with the underlying bulk band structure, they must keep connecting the bulk bands associated with the two TDPs. However, due to symmetry constraints, surface states cannot abruptly terminate; therefore, an intricate intermediate state must exist between the terminations of the two TDPs. The three-dimensional diagram illustrated in Figure 3(i) provides a visual description on the characteristics of variation of Fermi arcs around the type III TDP, where the red line in the middle shows the Fermi arc right at *ω*_TDP_.

**Figure 4:**
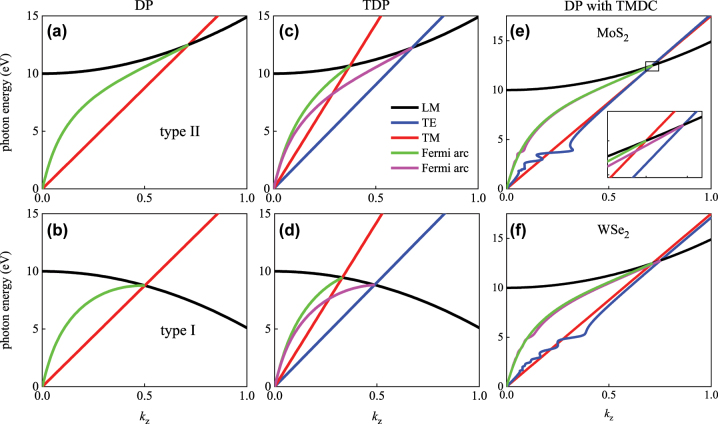
Fermi arcs with nonlocal effect: (a) type II (*α* = 0.001) and (b) type I (*α* = −0.001) DP; (c) type II and (d) type I TDP. (e) And (f): type II DP covered with TMDC monolayer, (e) MoS_2_, (f) MoSe_2_. The inset in (e) shows the detailed characteristics of band structure and corresponding Fermi arcs.

When the mutual interaction between the unit structures of metamaterials is strong enough, the generated nonlocal effect cannot be ignored. Such that we further investigate the behavior of Fermi arcs of DP and TDP with nonlocal effect. Since the high order nonlocal effects are much smaller than the second order nonlocality, here we consider a second order nonlocal effect on LM as $\varepsilon_{z}=\mu_{z}=1-{\left(\omega_{p}+\alpha k_{z}^{2}\right)}^{2}/\omega^{2}$ , where *α* is the nonlocal strength factor: for *α* < 0, LM endures negative dispersion, generate a type I DP; for *α* > 0, dispersion of LM is positive, corresponding to type II DP. With the nonlocal effect, locations of the two TDPs in the energy band respectively shift to $\omega =\frac{1-\sqrt{1-4\alpha \omega_{p}\varepsilon_{x,y}\mu_{1}}}{2\alpha \varepsilon_{x,y}\mu_{1}},k_{z}=\frac{1-\sqrt{1-4\alpha \omega_{p}\varepsilon_{x,y}\mu_{1}}}{2\alpha \sqrt{\varepsilon_{x,y}\mu_{1}}}$ , as shown in Figure 4(c) and (d); when *ɛ*_*x*_ = *ɛ*_*y*_, it returns to the DP configuration in Figure 4(a) and (b). We further obtain the dispersion of surface wave between air and the TDP medium with the nonlocal effect as:

(3) $$\begin{array}{ll} & \omega^{2}\sqrt{\left(k_{z}^{2}-\varepsilon_{x}\mu_{1}\omega^{2}\right)\left(k_{z}^{2}-\varepsilon_{y}\mu_{1}\omega^{2}\right)}\\ & \;+\sqrt{\varepsilon_{y}\mu_{1}}\left(\omega^{2}-k_{z}^{2}\right)\left(\alpha^{2}k_{z}^{4}+2\alpha \omega_{p}k_{z}^{2}+\omega_{p}^{2}-\omega^{2}\right)\\ & \;+\omega \sqrt{\omega^{2}-k_{z}^{2}}\sqrt{\alpha^{2}k_{z}^{4}+2\alpha \omega_{p}k_{z}^{2}+\omega_{p}^{2}-\omega^{2}}\\ & \;\times \left(\sqrt{\varepsilon_{y}\left(k_{z}^{2}-\varepsilon_{x}\mu_{1}\omega^{2}\right)}+\sqrt{\mu_{1}\left(k_{z}^{2}-\varepsilon_{y}\mu_{1}\omega^{2}\right)}\right)=0 \end{array}$$

Band structure and corresponding Fermi arcs of type I (*α* = −0.001) and type II (*α* = 0.001) DPs are shown in Figure 4(a) and (b). As a contrast, Fermi arcs of type I and type II TDPs are shown in Figure 4(c) and (d). It shows for TDP, the two coincident Fermi arcs of DP splits into two separate ones, and all the Fermi arcs exactly end on the type I and type II DPs and TDPs. Tuning the Fermi arcs with nonlocal effect by 2D TMDC materials is also investigated, the analytical expression of surface wave of DP with nonlocal effect and simultaneously with TMDC surface conductivity is obtained as:

(4) $$\begin{array}{cc} & \alpha^{2}\sqrt{\varepsilon_{1}\mu_{1}}k_{z}^{4}\left(\omega^{2}-k_{z}^{2}\right)+2\alpha \sqrt{\varepsilon_{1}\mu_{1}}k_{z}^{2}\left(\omega^{2}-k_{z}^{2}\right)+\sqrt{\varepsilon_{1}\mu_{1}}k_{z}^{2}\left(\omega^{2}-\omega_{p}^{2}\right)\\ & \;=\omega \left\{\begin{array}{c} \sqrt{k_{z}^{2}-\varepsilon_{1}\mu_{1}\omega^{2}}\left(-\omega \sqrt{k_{z}^{2}+\mu_{1}\omega \left(i\sigma -\varepsilon_{1}\omega \right)}-\sqrt{\mu_{1}}\sqrt{\omega^{2}-k_{z}^{2}}\sqrt{\alpha^{2}k_{z}^{4}+2\alpha \omega_{p}k_{z}^{2}+\omega_{p}^{2}-\omega^{2}}\right)\\ +\sqrt{\varepsilon_{1}}\left[\sqrt{\mu_{1}}\omega \left(\omega^{2}-\omega_{p}^{2}\right)-\sqrt{\omega^{2}-k_{z}^{2}}\sqrt{k_{z}^{2}+\mu_{1}\omega \left(i\sigma -\varepsilon_{1}\omega \right)}\sqrt{\alpha^{2}k_{z}^{4}+2\alpha \omega_{p}k_{z}^{2}+\omega_{p}^{2}-\omega^{2}}\right] \end{array}\right\} \end{array}$$

Band structure and Fermi arcs of type II DP covered by MoS_2_ and MoSe_2_ monolayer are respectively shown in Figure 4(e) and (f), the inset in Figure 4(e) shows the detailed feature. It shows the two Fermi arcs also completely terminate on TDPs that induced by the surface conductivity of TMDC. It demonstrates that the 2D TMDC also provides an effective approach for effective tuning the Fermi arcs with nonlocal effect.

**Figure 5:**
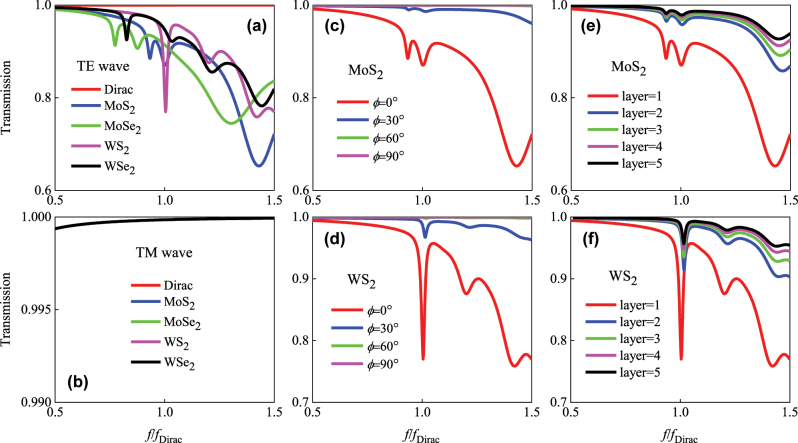
Modulation of transmission of PDS by covering 2D TMDC sheets: (a) and (b) covered with different TMDC monolayer, incident by TE and TM waves, respectively; (c) and (d) respectively covered by MoS_2_ and WSe_2_ monolayer with the variation of angle between LM of PDS and *y* axis, incident by TE wave; (e) and (f) covered by different layers of MoS_2_ and WSe_2_ sheets, respectively, incident by TE wave.

In view of the exceptional properties of 2D TMDC materials, it is instructive to investigate its function on the transportation characteristics of DP medium. Configurations for calculating the transmission of PDS are as follows: the length of PDS is set 50 μm and the other two dimensions are infinite in size. Calculated transmissions of PDS with covered TMDC sheets are shown in Figure 5, where the DP frequency is set to 2 eV that on the resonance of TMDC. As shown in Figure 5(a) and (b), by covering various TMDC monolayer, transmission spectra of TE wave exhibit a series of resonant peaks that are generated by the excitons associated with interband transitions at the *K* point in the Brillouin zone of TDMC [30], which show more attenuation in the high frequency band. While the transmissions of TM wave still maintain a broad band high transmission around Dirac point as shown in Figure 5(b), since the surface conductance of TMDC monolayer mainly acts on the TE modes.

By tuning the relative angle *ϕ* between LM of DP medium and *y* axis, transmissions can be further modulated. As respectively shown in Figure 5(c) and (d), with the rotation angle *ϕ* increasing, transmissions of TE wave decrease sharply, while the main oscillating features of the covered MoS_2_ and WSe_2_ monolayer are kept, where the sharp dip of resonance in the transmission spectra could be used for accurate frequency detection. Another controlling freedom is the layers of TMDC sheets. As respectively shown in Figure 5(e) and (f), with the layers of covered MoS_2_ and WSe_2_ sheets increasing 1–5, transmissions decrease gradually due to the declining surface conductance, and eventually stabilizes when layer number = 5. It shows the 2D TMDC materials could also modulate the transmissions of PDS in multi-freedoms. In the experimental measurement, surface wave and transmissions of TMDC sheets coated PDS can be measured via scanning near-field optical microscopy (SNOM), polarimetry, and angular spectroscopy [31]. The corresponding Fermi arcs can be obtained by applying Fourier transform of the measured surface wave distribution. Future work could explore dynamical modulation of Dirac states using TMDC’s phase-change properties.

In addition to the topological degeneracy point and Fermi arc, morphology of EFC of the type III TDP system is comparably interesting and important, since the electromagnetic density of state (DOS) and scattering cross section (SCS) strongly depends on the transformation of EFC. Eigen-frequency of the type III TDP can be derived by solving Eq. (2) as

(5) $$\omega =\frac{1}{\sqrt{\varepsilon_{x}\mu_{1}}}\left(k_{z}\pm \sqrt{\frac{\varepsilon_{x}}{\varepsilon_{z}}{k_{x}}^{2}+\frac{\mu_{1}}{\mu_{z}}{k_{y}}^{2}+{k_{z}}^{2}}\right)$$

**Figure 6:**
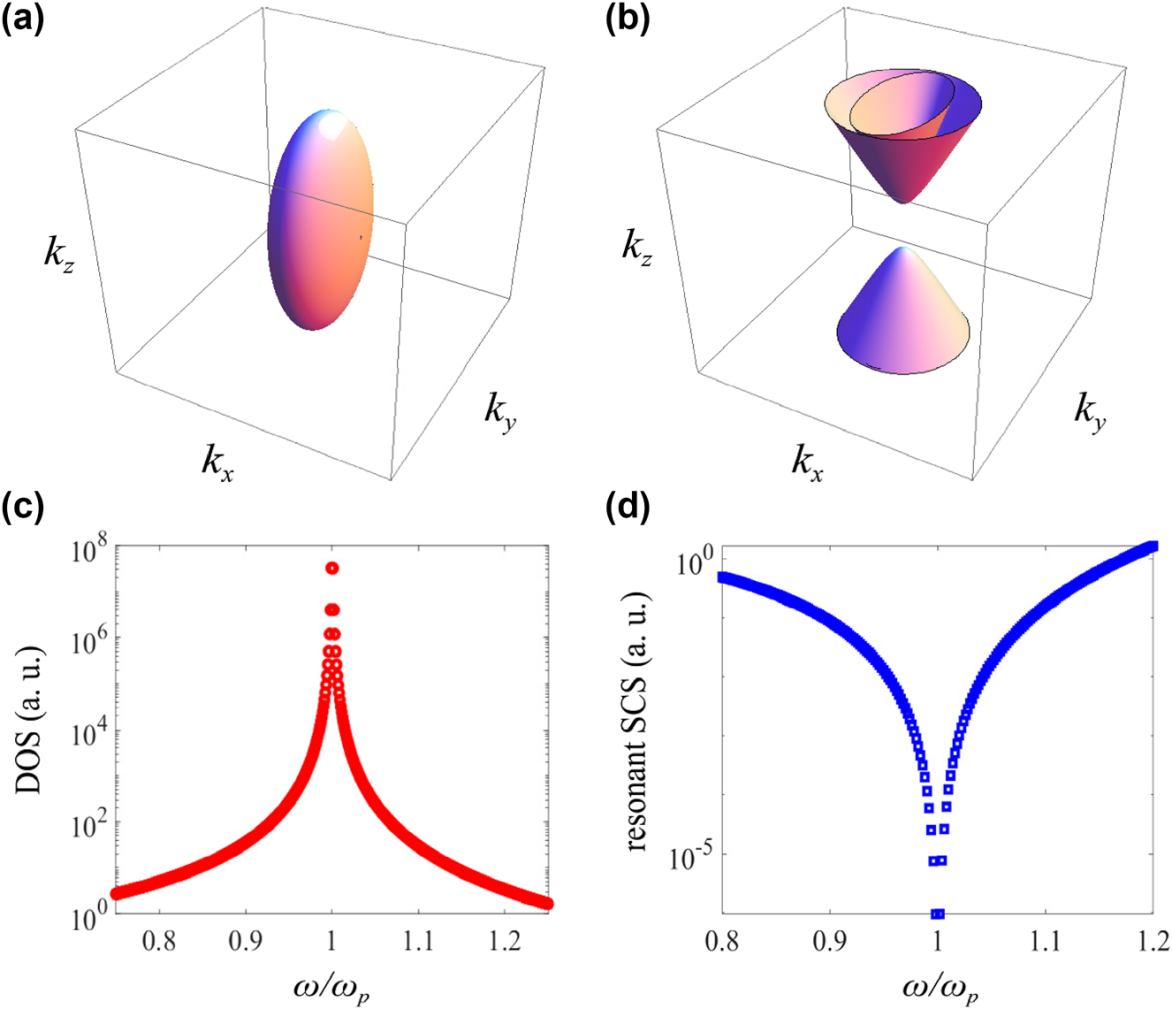
EFC and DOS of type III TDP. (a) and (b) EFC of type III TDP below and above the TDP frequency; (c) and (d) variations of DOS and resonant SCS around the TDP frequency.

Corresponding structures of EFC of type III TDP is shown in Figure 6. Interestingly, when *ω* < *ω*_*TP*_, EFC is elliptical [Figure 6(a)], while when *ω* > *ω*_*TP*_, EFC is hyperbolic [Figure 6(b)]. It means that transformation of EFC with frequency should endure a topological transition at TDP, which can be considered as an analogue of Lifshitz transition in electronic band structures and simultaneously experiences discontinuities and anomalies in DOS, it plays a crucial role in the characteristics of topological semimetals. Consequently, it is expected that the DOS and SCS of TDP system should exhibit specific behavior around *ω*_TDP_. Variation of $\text{DO}{\text{S}}_{\text{TDP}}=\int \int_{-\infty }^{\infty }1/\left|v\right|d^{2}k$ $\text{DO}{\text{S}}_{\text{TDP}}=\iint 1/\left|v\right|d^{2}k$ around *ω*_TDP_ is shown in Figure 6(c), where **v** is the group velocity, the integral bound of wavevector is not truncated. As it shows, when approaching *ω*_TDP_, DOS of TDP system increases exponentially and finally diverges at *ω*_TDP_ due to the topological transition of EFC at TDP.

Another remarkable feature of the type III TDP system is the resonant SCS of photons around *ω*_TDP_. When the transition frequency of the embedded two-level atom resonates with the surrounding TDP medium, the resonant SCS solely depends on the transformation of the area of EFC [32], [33], [34]. As shown in Figure 6(d), resonant SCS decreases exponentially when the frequency approaching *ω*_TDP_, and declines to the minimum at *ω*_TDP_. The underlying physical mechanism can be revealed by the transformation of EFC of TDP: when the frequency approaches *ω*_TDP_ from either below or above *ω*_TDP_, the elliptical or hyperbolic EFC shrinks and becomes extremely narrow at *ω*_TDP_, which results in tremendous large surface area of EFC with multi-eigenmode channels in the momentum space, that leads to the extreme values and topological transitions of DOS and resonant SCS at *ω*_TDP_ [35], [36]. The observed topological transition from open (hyperbolic-like) to closed (elliptical-like) EFC, diverge DOS and diminishing SCS at type III TDP may provide potential applications such as EFC engineering.

## 4 Conclusions

In conclusion, we theoretically demonstrate the modulations of Fermi arcs of DP in multi-degrees of freedom by 2D TMDC materials. Interestingly, Fermi arcs of type III TDP exhibit unconventional behavior that may do not terminate at degeneracy point. Multiple modulations of transmissions of PDS by TMDC layers are also studied. Fermi arcs with nonlocal effect are analyzed. We also manifest the topological transitions of EFC of type III TDP. Our finding may provide potential applications in modulations of Fermi arcs with feasibility and enriches the classification of Fermi arcs.

## Appendix A: Derivation of the analytical dispersion of surface waves of TDP

Assuming that the interface is along the *x*–*z* plane, where the air locates in the upper half-space with positive *y* and the DP medium locates in the lower half-space with negative *y*. Such that the surface wave decays along *y* direction. By solving the Maxwell equations, two orthogonal eigenstate electromagnetic wave of DP medium with covered TMDC layers are obtained as

(A1)
$$\begin{array}{c} \psi_{TP}^{1}=\left[\begin{array}{cccccc} -i\sqrt{\frac{\mu_{1}\left(\omega^{2}-\omega_{p}^{2}\right)}{k_{z}^{2}+\mu_{1}\omega \left[i\sigma \left(\omega \right)-\omega \varepsilon_{1}\right]}} & 0 & 0 & 0 & -i\frac{k_{z}}{\omega }\sqrt{\frac{\omega^{2}-\omega_{p}^{2}}{\mu_{1}\left\{k_{z}^{2}+\mu_{1}\omega \left[i\sigma \left(\omega \right)-\omega \varepsilon_{1}\right]\right\}}} & 1 \end{array}\right]\\ \psi_{TP}^{2}=\left[\begin{array}{cccccc} 0 & -\frac{k_{z}}{\varepsilon_{1}\omega } & -i\sqrt{\frac{k_{z}^{2}-\varepsilon_{1}\mu_{1}\omega^{2}}{\varepsilon_{1}\left(\omega^{2}-\omega_{p}^{2}\right)}} & 1 & 0 & 0 \end{array}\right] \end{array}$$

where the eigenstates take the forms of $\left[\begin{array}{cccccc} E_{x} & E_{y} & E_{z} & H_{x} & H_{y} & H_{z} \end{array}\right]$ . And the two orthogonal eigenstate electromagnetic wave (TE/TM mode) of air are

(A2)
$$\begin{array}{c} \psi_{\mathrm{air}}^{1}=\left[\begin{array}{cccccc} \frac{i\omega }{\sqrt{k_{z}^{2}-\omega^{2}}} & 0 & 0 & 0 & \frac{ik_{z}}{\sqrt{k_{z}^{2}-\omega^{2}}} & 1 \end{array}\right]\\ \psi_{\mathrm{air}}^{2}=\left[\begin{array}{cccccc} 0 & -\frac{k_{z}}{\omega } & \frac{i\sqrt{k_{z}^{2}-\omega^{2}}}{\omega } & 1 & 0 & 0 \end{array}\right] \end{array}$$

The existing surface wave at the interface is a mixture of the above four modes wave: TE/TM modes in the air and the two eigenwaves in the DP medium with covered TMDC sheets. We then followed the approach discovered by Dyakonov, applying the boundary condition that the tangential components of electric field and magnetic field are matched at the interface, dispersion of the surface wave can be obtained as Eq. (1) in the maintext.

## Appendix B: Effective Hamiltonian of type III triple degeneracy point

A Hermitian Hamiltonian describing such electromagnetic type III triple degeneracy point (TDP) can be obtained through Maxwell equations as

(A3)
$$H_{0}\psi =N^{-1/2}MN^{-1/2}\psi =EN^{-1/2}\psi$$

where

(A4)
$$M=\left(\begin{array}{cccccccc} 0 & 0 & 0 & 0 & k_{z} & -k_{y} & 0 & 0\\ 0 & 0 & 0 & -k_{z} & 0 & k_{x} & 0 & 0\\ 0 & 0 & 0 & k_{y} & -k_{x} & 0 & i/\omega_{p} & 0\\ 0 & -k_{z} & k_{y} & 0 & 0 & 0 & 0 & 0\\ k_{z} & 0 & -k_{x} & 0 & 0 & 0 & 0 & 0\\ -k_{y} & k_{x} & 0 & 0 & 0 & 0 & 0 & i/\omega_{p}\\ 0 & 0 & -i/\omega_{p} & 0 & 0 & 0 & 0 & 0\\ 0 & 0 & 0 & 0 & 0 & -i/\omega_{p} & 0 & 0 \end{array}\right)$$

and

(A5)
$$N=\left(\begin{array}{cccccccc} \varepsilon_{x} & 0 & 0 & 0 & 0 & 0 & 0 & 0\\ 0 & \varepsilon_{y} & 0 & 0 & 0 & 0 & 0 & 0\\ 0 & 0 & \varepsilon_{z} & 0 & 0 & 0 & 0 & 0\\ 0 & 0 & 0 & u_{1} & 0 & 0 & 0 & 0\\ 0 & 0 & 0 & 0 & u_{1} & 0 & 0 & 0\\ 0 & 0 & 0 & 0 & 0 & u_{z} & 0 & 0\\ 0 & 0 & 0 & 0 & 0 & 0 & 1/\varepsilon_{z} & 0\\ 0 & 0 & 0 & 0 & 0 & 0 & 0 & 1/u_{z} \end{array}\right)$$

where we set *ɛ*_0_ = *μ*_0_ = 1, and the base eigenvector is

(A6)
$$\psi =\left[\begin{array}{cccccccc} E_{x} & E_{y} & E_{z} & H_{x} & H_{y} & H_{z} & dP_{z}/dt & dM_{z}/dt \end{array}\right]$$

where *P* and *M* are electric and magnetic dipole moments, respectively. By solving the above Schrodinger equation, eigenvectors of the TDP can be obtained as

(A7)
$$\begin{array}{c} \left|\psi_{1}\right\rangle =\left[\begin{array}{cccccccc} \frac{1}{\sqrt{\varepsilon_{x}\;}} & 0 & 0 & 0 & \frac{1}{\sqrt{\;\mu_{x}}} & 0 & 0 & 0 \end{array}\right]\\ \left|\psi_{2}\right\rangle =\left[\begin{array}{cccccccc} 0 & 0 & \frac{i}{\omega_{p}\;\sqrt{\varepsilon_{z}}} & 0 & 0 & 0 & \sqrt{\varepsilon_{z}} & 0 \end{array}\right]\\ \left|\psi_{3}\right\rangle =\left[\begin{array}{cccccccc} 0 & 0 & 0 & 0 & 0 & \frac{i}{\omega_{p}\;\sqrt{\mu_{z}}} & 0 & \sqrt{\mu_{z}} \end{array}\right] \end{array}$$

Then the effective Hamiltonian of the TDP can be derived by using the *k*·*p* method $H_{ij}^{TP}=\left\langle \psi_{i}\right|H_{0}\left|\psi_{j}\right\rangle$ as

(A8)
$$\begin{array}{ll} H_{TP} & =\left(\begin{array}{ccc} \frac{2k_{z}}{\sqrt{\varepsilon_{x}\mu_{x}}} & -\frac{ik_{x}}{\sqrt{\varepsilon_{z}\mu_{x}}} & -\frac{ik_{y}}{\sqrt{\varepsilon_{x}\mu_{z}}}\\ \frac{ik_{x}}{\sqrt{\varepsilon_{z}\mu_{x}}} & 0 & 0\\ \frac{ik_{y}}{\sqrt{\varepsilon_{x}\mu_{z}}} & 0 & 0 \end{array}\right)\\ & =c_{1}k_{z}I+\left(\begin{array}{ccc} c_{2}k_{z} & -\frac{ik_{x}}{\sqrt{\varepsilon_{z}\mu_{x}}} & -\frac{ik_{y}}{\sqrt{\varepsilon_{x}\mu_{z}}}\\ \frac{ik_{x}}{\sqrt{\varepsilon_{z}\mu_{x}}} & -c_{2}k_{z} & 0\\ \frac{ik_{y}}{\sqrt{\varepsilon_{x}\mu_{z}}} & 0 & -c_{2}k_{z} \end{array}\right)\\ c_{1} & =c_{2}=\frac{1}{\sqrt{\varepsilon_{x}\mu_{x}}} \end{array}$$

Therefore, the obtained TDP is a type III TDP.
